# Short-term renal and metabolic effects of low dose vildagliptin treatment added-on insulin therapy in non-proteinuric patients with type 2 diabetes: open-label randomized prospective study

**DOI:** 10.20945/2359-3997000000220

**Published:** 2020-03-30

**Authors:** Valentina K. Bayrasheva, Ivan Y. Pchelin, Vladimir A. Dobronravov, Alina Yu. Babenko, Svetlana G. Chefu, Ivan S. Shatalov, Volha N. Vasilkova, Natalia V. Hudiakova, Alexandra N. Ivanova, Pavel A. Andoskin, Elena N. Grineva

**Affiliations:** 1 Institute of Endocrinology Almazov National Medical Research Centre Saint Petersburg Russia Institute of Endocrinology , Almazov National Medical Research Centre , Saint Petersburg , Russia; 2 Department of Faculty Therapy Saint Petersburg State University, Saint Petersburg Russia Department of Faculty Therapy , Saint Petersburg State University, Saint Petersburg , Russia; 3 Research Institute of Nephrology Pavlov First Saint Petersburg State Medical University Saint Petersburg Russia Research Institute of Nephrology , Pavlov First Saint Petersburg State Medical University , Saint Petersburg , Russia; 4 Department of Faculty Therapy, Pavlov First Saint Petersburg State Medical University, Saint Petersburg Russia Department of Faculty Therapy, Pavlov First Saint Petersburg State Medical University, Saint Petersburg , Russia; 5 Experimental Research Laboratory Laser Medicine Center Pavlov First Saint Petersburg State Medical University Saint Petersburg Russia Experimental Research Laboratory of the Laser Medicine Center , Pavlov First Saint Petersburg State Medical University , Saint Petersburg , Russia; 6 Scientific and Research Institute of Bioengineering Saint Petersburg National Research University of Information, Technologies, Mechanics and Optics Saint Petersburg Russia Scientific and Research Institute of Bioengineering , Saint Petersburg National Research University of Information, Technologies, Mechanics and Optics , Saint Petersburg , Russia; 7 Department of Internal Medicine No.1 with the Course of Endocrinology Gomel State Medical University Gomel Belarus Department of Internal Medicine No.1 with the Course of Endocrinology , Gomel State Medical University , Gomel , Belarus; 8 Laboratory of Protein Biochemistry State Research Institute of Highly Pure BioSubstances Saint Petersburg Russia Laboratory of Protein Biochemistry , State Research Institute of Highly Pure BioSubstances , Saint Petersburg , Russia

**Keywords:** Vildagliptin, type 2 diabetes, renal function, NGAL, cystatin C, type IV collagen

## Abstract

**Objective:**

The aim of this randomized comparative study was to assess renal and metabolic effects of vildagliptin in insulin-treated type 2 diabetes (T2DM) patients without overt chronic kidney disease.

**Subjects and methods:**

We randomized 47 insulin-treated non-proteinuric patients with satisfactory controlled T2DM and estimated glomerular filtration rate (eGFR) ≥ 60 mL/min/1.73m ^2^ either to continue insulin therapy (control) or to receive combined insulin-vildagliptin treatment (VIG group). We assessed eGFR using serum creatinine (eGFRcreat), cystatin C (eGFRcys), and both (eGFRcreat-cys), and urinary creatinine-adjusted excretion of albumin (UACR), type IV collagen (uCol IV/Cr), and neutrophil gelatinase-associated lipocalin (uNGAL/Cr) at baseline and after 6 months of treatment.

**Results:**

Study groups were comparable in terms of age and sex (60.1 ± 6.1 years and 42.9% men in control group vs. 60.8 ± 5.2 years and 39.1% in VIG group). After 6 months of treatment, there were no significant changes in main assessed parameters in control group. VIG group demonstrated significant decrease in HbA1c, diastolic blood pressure, frequency of hypoglycemia, and high-sensitivity C-reactive protein level as compared to the changes in control group. While eGFRcreat, UACR, and uNGAL/Cr showed no significant changes after vildagliptin addition, eGFRcys, eGFRcreat-cys, and uCol IV/Cr changed significantly in comparison with control group (+7.0% [3.7;13.3]; +5.1% [1.4;8.5]; -32,8% [-55.8;-24.4], respectively, p < 0.01 each). Correlation and regression analysis revealed glucose-independent pattern of these changes.

**Conclusion:**

Addition of vildagliptin to ongoing insulin therapy in patients with T2DM was associated with a reduction in uCol IV/Cr and an increase in eGFRcys and eGFRcreat-cys, independent of T2DM control parameters.

## INTRODUCTION

Recently, a wide range of effective hypoglycemic agents has become available for the treatment of type 2 diabetes mellitus (T2DM) (1). Nevertheless, many patients with adequate glycemic control develop diabetic nephropathy (DN) (2,3). Thus, investigation of renal pleiotropic effects of novel antidiabetic medications could be crucial for improving treatment strategies and prognosis of chronic kidney disease in T2DM (DM-CKD) (4).

Dipeptidyl peptidase 4 (DPP-4) inhibitors have been shown to exert some beneficial renal effects (5,6). Renal effects of DPP-4 inhibitors result from activation of renal GLP-1 receptors and incretin-independent actions (5-7). GLP-1-independent pleiotropic effects may be mediated by the changes in the blood levels of various enzyme substrates with natriuretic, anti-inflammatory, antioxidative, vasodilating, and immunomodulatory properties (5-10). Moreover, DPP-4 inhibitors could directly affect renal structures with DPP-4 expression (proximal tubules and glomerular capillaries) (5-7,11,12). However, it is not quite clear whether DPP-4 inhibitors with predominant renal elimination pathway (sitagliptin, alogliptin, vildagliptin) (13) have advantages in the setting of DN.

Renoprotective potential of vildagliptin has been demonstrated by several studies using cell culture (14) and experimental models of diabetic and non-diabetic renal dysfunction (15-17). Nevertheless, clinical data on beneficial renal effects of vildagliptin are limited (18-21). Several pilot studies demonstrated that short-term addition of vildagliptin to different oral antidiabetic medications resulted in a significant decrease in urinary albumin-to-creatinine ratio (UACR) without any changes in glomerular filtration rate (GFR) estimated using creatinine (eGFRcreat) (19,21). Moreover, this effect was observed in patients with early stages of DN, but not in proteinuric patients (19). However, all the aforementioned studies were conducted without any control groups. Thus, they can’t account for the influence of spontaneous reduction in albuminuria, which, due to the specificities of natural progression of DN, is possible in 30% patients (22). Furthermore, the influence of satisfactory metabolic control on the realization of pleiotropic effects of vildagliptin remains unknown. It is also unclear whether vildagliptin has renal benefits in insulin-treated patients, since insulin requirement could result from progressive deterioration of its endogenous secretion as a consequence of impaired response to incretins (23).

The aim of the present study was to assess renal and metabolic effects of vildagliptin in insulin-treated non-proteinuric T2DM patients with satisfactory glycemic and blood pressure control. To assess preclinical renal changes under vildagliptin administration we investigated urinary excretion of type IV collagen (uCol IV) and tubular injury marker neutrophil gelatinase-associated lipocalin (uNGAL) that are known to be increased prior to the development of albuminuria in patients with DM (24,25). For evaluation of renal function we used CKD-EPI cystatin C-containing equations that are considered to be more precise than creatinine-based estimation methods, especially for individuals without a significant decline in GFR (26).

## SUBJECTS AND METHODS

### Patients

The study was approved by the Ethics Committee of Almazov National Medical Research Centre and was performed in accordance with the principles of the Declaration of Helsinki. All patients provided written informed consent to participate in the study. This was a single-center, open-label, non-blinded, randomized comparative clinical study.

Inclusion criteria were: male and female Caucasians with T2DM; age between 45 and 70 years; insulin treatment in basal-bolus or basal plus regimens; glycated hemoglobin (HbA1c) and blood pressure (BP) within the target range or close to individual target levels; eGFRcreat ≥ 60 mL/min/1.73 m ^2^ ; mixed type of nutrition (not vegetarian); urinary albumin concentration (morning spot) < 300 mg/L confirmed in at least two out of three consecutive morning spot urine samples. Non-diabetic causes of CKD among patients with abnormal urinary albumin concentration were ruled out by ultrasonography, urine culture and microscopy analysis.

Non-inclusion criteria were: hypersensitivity to any component of vildagliptin-containing tablets; patient’s noncompliance and nonadherence; non-diabetic kidney disease or urine sediment abnormalities, prostatic hyperplasia with obstructive symptoms; severe micro- and macrovascular diabetic complications (including proliferative diabetic retinopathy, diabetic foot ulcer and neuropathic osteoarthropathy; recent acute coronary syndrome, myocardial infarction, or other cardiovascular events (less than 6 months ago); recent serious acute events including surgical interventions (less than 6 months ago); severe stable cardiac angina, clinically advanced congestive heart failure (NYHA III-IV); planned surgical intervention; uncontrolled dyslipidemia; elevation in ALT/AST level exceeding two-fold the upper limit of normal; nephrotoxic drugs intake; morbid obesity with body mass index (BMI) ≥ 40 kg/m ^2^ ; moderate and severe anemia; thyroid dysfunction; systemic autoimmune disorders; chronic infection; malignancy; immunosuppressive therapy and regular nonsteroidal anti-inflammatory drugs intake; pregnancy; lactation.

Patients with a history of arterial hypertension and other cardiovascular diseases continued to receive previously assigned antihypertensive, lipid lowering, antiplatelet therapy, and beta blockers, as appropriate.

We enrolled and randomized (by computer-generated random number) 47 patients aged 49-70 years with T2DM either to continue insulin therapy (control insulin group, n = 23) or to receive vildagliptin in a daily dose of 50 mg added-on insulin (vildagliptin-insulin group (VIG), n = 24) for 6 months. Three patients were lost to follow-up. Reasonable efforts were made to ascertain the reasons for their withdrawing. The patients reported no side effects or adverse events. They opted to withdraw from the study for family reasons or because they moved away from the study site.

At baseline patients underwent detailed assessment of medical and current medication history including analysis of daily insulin doses and the frequency of hypoglycemia. Hypoglycemia was detected as any symptomatic event with or without blood glucose measurement, or confirmed hypoglycemia with plasma glucose level of ≤ 3.9 mmol/L with or without symptoms (27). Diabetic retinopathy was graded according to the Early Treatment Diabetic Retinopathy Study protocol. Diabetic neuropathy was diagnosed using the Michigan Neuropath Screening Instrument questionnaire and clinical examination of both feet including visual inspection, grading of ankle reflexes, semiquantitative assessment of vibration perception at the great toe, temperature perception by Tiptherm, and 10 g monofilament testing.

### Measurements

We investigated the effects of six-month vildagliptin and insulin combined therapy (in comparison with insulin monotherapy) on the changes in the levels of renal biomarkers (eGFR based on serum creatinine (eGFRcreat), cystatin C (eGFRcys), and both (eGFRcreat-cys), UACR, urinary creatinine-adjusted excretion of type IV collagen (uCol IV/Cr) and NGAL (uNGAL/Cr)). We also evaluated glycemic control parameters (fasting and postprandial glycemia, HbA1c, frequency of hypoglycemic episodes), metabolic parameters (BMI, serum cholesterol and triglycerides), systolic and diastolic BP, and high-sensitivity C-reactive protein (hsCRP).

Average values of last three measured levels of fasting and postprandial (two hours after breakfast) glycemia were taken into account. Blood pressure (BP) measurements were taken twice in 10 min interval after 5 min of rest using Omron M3 Expert tonometer (Kyoto, Japan). Average value of two measurements was taken into account.

All patients received recommendations not to change water, meat, and protein intake, and to avoid strenuous physical exercise 3 days prior to the collection of blood and urine samples.

### Study end points

The primary study end points included a statistically significant increase in eGFRcys and eGFRcreat-cys and a reduction in UACR. Secondary outcomes included statistically significant changes in the levels of urinary markers and BP from baseline. Tertiary confirmatory end points comprised changes in HbA1c, frequency of hypoglycemic episodes, lipids, and body weight.

### Assessment of laboratory parameters

HbA1c was measured by high-performance liquid chromatography method using BioRad commercial kit (Hercules, USA) on HPLC-analyzer BioRad d10 (Hercules,USA).

Total cholesterol, triglycerides, serum and urinary creatinine, urinary albumin, hsCRP, and serum cystatin C were measured using appropriate commercial kits by Cobas Integra, Roche (Mannheim, Germany) on clinical chemistry analyzer Cobas Integra 400 plus (Roche Diagnostics, Indianapolis, USA). Creatinine was measured by enzymatic method traceable to isotope dilution mass spectrometry reference. hsCRP and serum cystatin C were assessed by immunoturbidimetric method. Urinary albumin was measured by the biuret reaction. eGFRcreat, eGFRcys, and eGFRcreat-cys were calculated using the Chronic Kidney Disease Epidemiology Collaboration (CKD-EPI) equation based on serum creatinine, cystatin C, and both, respectively (26).

Quantitative analysis of uCol IV in urine samples collected in glass tubes with stabilization buffer (BIO84, Argutus Medical, Dublin, Ireland) was performed by enzyme-linked immunosorbent assay, using the protocol of ELISA-kit for collagen type IV assessment by Argutus Medical (Dublin, Ireland) on ELx800 Absorbance microplate reader (Winooski, VT, USA). uNGAL was assayed by immunochemiluminescent method using Abbott Architect i2000analyzer (Santa Clara, USA).

### Statistical analysis

Calculation of power and sample size showed that 22 patients completing the study could provide 80% power to demonstrate a significant difference between two groups in hypoalbuminuric effect if the true difference was 15%. This was based on the assumption that intra-individual coefficient of variation for UACR was 10%. ROC curves analysis was used to assess the diagnostic accuracy and the optimal cut-off for the urinary markers (uNGAL/Cr: sensitivity – 73.1%, specificity – 70.4%, optimal cut-off – 8.45 μg/g Cr., p < 0,001; uCol IV/Cr: 73.1%, 65.4%, and 2.65 μg/g Cr., respectively, p < 0,001). The proportions of patients were compared using the chi-square test. The baseline levels of biomarkers and the dynamics of assayed parameters in the groups were compared using Mann-Whitney U-test. Wilcoxon rank sum test was used to analyze intra-group changes. Repeated measures in the General Linear Models were used to analyze the intergroup differences in the observed changes. The results were expressed as median [25th percentile; 75th percentile]. Relationships between significant changes in renal biomarkers and changes in potential explanatory variables (glycemic control and metabolic parameters, BP, hsCRP) were investigated by Spearman’s correlation. Stepwise linear regression included the dynamics of significantly changed renal biomarkers as dependent variables. The changes in glycemic control and metabolic parameters, BP, and hsCRP, as well as categorical determinants (smoking, history of cardiovascular disease, use of antihypertensive and lipid-lowering medications), were taken as independent variables. β denoted standardized regression coefficients and R ^2^ – the coefficient of determination. p-values < 0.05 were considered significant. Statistical analysis was performed using IBM SPSS Statistics version 22.0 (New York, USA).

## RESULTS

### Clinical characteristics of the study groups

Baseline clinical and laboratory features are shown in Table 1 . In general, there were no significant differences in baseline clinical and laboratory parameters between the groups ( Table 1 ).

**Table 1:** Baseline clinical characteristics of patients with T2DM in the studied groups. Data are reported either as the median [25th percentile; 75th percentile] or as a raw number (percentage) within the study group. The p-values are from either Mann-Whitney U-tests or from chi-square tests for categorical variables

Variable	Insulin-treated group (Control) n = 21	Insulin-vildagliptin-treated group (VIG) n = 23	P
Age (years)	60.0 [54.0; 66.0]	62.0 [58.0; 63.0]	0.56
Male, n (%)	9 (42.9%)	9 (39.1%)	0.80
Known diabetes duration (years)	10.0 [6.0; 13.0]	8.0 [7.0; 12.0]	0.72
Fasting blood glucose, mmol/L	6.4 [5.8; 7.4]	6.9 [6.5; 7.3]	0.08
Postprandial blood glucose, mmol/L	7.9 [7.3; 8.9]	8.2 [7.9; 9.1]	0.38
HbA1c, %	7.2 [6.6; 7.6]	7.3 [6.9; 7.6]	0.55
Daily insulin dose, units/kg	0.44 [0.37; 0.63]	0.43 [0.37; 0.54]	0.76
Frequency of hypoglycemic episodes (per month)	1.0 [0.3; 2.0]	1.0 [0.5; 2.0]	0.87
Diabetic neuropathy, n (%)	17 (81.0%)	19 (82.6%)	0.89
Diabetic retinopathy, n (%)	10 (47.6%)	9 (39.1%)	0.57
Diabetic nephropathy, CKD A2 category, n (%)	10 (47.6%)	12 (52.2%)	0.76
BMI, kg/m ^2^	31.5 [28.0; 35.4]	31.6 [30.3;34.9]	0.56
Total cholesterol, mmol/L	5.4 [4.8; 6.3]	5.5 [4.8; 6.1]	0.81
Triglycerides, mmol/L	1.5 [1.3; 1.9]	1.6 [1.1; 1.9]	0.82
hsCRP, mg/L	3.62 [1.54; 5.40]	2,73 [0.84; 5.20]	0.43
Lipid-lowering medication intake, n (%): – statins – fibrates	11 (52.4%) 10 (47.6%) 0 1 (4.8%)	15 (65.2%) 12 (52.2%) 2 (8.7%) 1 (4.3%)	0.93
Systolic blood pressure, mmHg	132 [125; 139]	133 [128; 138]	0.56
Diastolic blood pressure, mmHg	78 [73; 84]	81 [75; 85]	0.25
Antihypertensive therapy intake, n (%)	17 (81.0%)	20 (86.9%)	0.59
RAS-modulating drugs intake, n (%)	16 (76.2%)	19 (82.6%)	0.60
Clinical manifestation of atherosclerosis, n (%) – ischemic heart disease – a history of myocardial infarction –a history of stroke – extracoronary atherosclerosis	11 (52.4%) 7 (33.3%) 3 (14.3%) 3 (14.3%) 9 (42.8%)	11 (47.8%) 7 (30.4%) 2 (8.7%) 1 (4.3%) 8 (35.0%)	0.76
Smoking, n (%)	7 (33.3%)	6 (26.1%)	0.59

RAS: renin-angiotensin system; hsCRP: high-sensitivity C-reactive protein; CKD: chronic kidney disease.

The majority of patients (more than 60%) had obesity ( Table 1 ). The prevalence of arterial hypertension was more than 80% in both groups. All patients with arterial hypertension received antihypertensive therapy, including angiotensin-converting enzyme inhibitors or angiotensin II receptor blockers. There was no statistical difference in the prevalence of clinical manifestations of atherosclerosis between the groups under similar parameters of systemic hemodynamics and serum lipid profile. Almost half of patients in both groups had moderately increased albuminuria (category A2 of CKD). The vast majority of the studied patients received glargine as a basal insulin and rapid acting insulin analogs for bolus insulin replacement, and there were no significant differences in insulin regimens between the two groups.

### The dynamics of metabolic and glycemic control parameters, blood pressure and hsCRP

In the insulin monotherapy group, baseline laboratory parameters showed no significant changes after 6 months of study. In contrast, in the VIG group we observed a significant decrease (from initial values) in HbA1c (by 2.8% [-4.5; -1.4], p = 0.002), fasting and postprandial blood glucose levels (by 5.2% [-11.4; -1.4], p = 0.006, and by 5.3% [-15.8; -1.3], p = 0.036, respectively). Furthermore, the addition of vildagliptin in patients with near-targeted values of HbA1c resulted in a significant reduction in the daily dose of insulin (by 6.5% [-10.0; -2.6] from initial, p = 0.001) and the incidence of hypoglycemic episodes (by 50.0% [-75.5; 12.5] from baseline value, p = 0.001). A significant reduction in diastolic arterial BP (from 78 mmHg [73; 84] to 74 mmHg [71; 82], p <0.001) and an inconsiderable reduction in systolic BP (-1.0 mmHg [-3.0; 2.0] from baseline value, p = 0.32) following the addition of vildagliptin to treatment were documented. hsCRP level was reduced significantly in the VIG group (-14.3% [-28.9; 17.2] from baseline value, p = 0.027), whereas no significant changes were noted in the control group.

Our study also demonstrated a mild reduction in serum level of triglycerides and, to a lesser degree, in median total cholesterol level in both groups, more pronounced in the VIG group. Relative (percentage) changes in the level of HbA1c, the frequency of hypoglycemic episodes, the values of hsCRP and diastolic BP, as well as reduction in the daily dose of insulin, were significantly different between the groups by the end of the study.

### Changes in the levels of evaluated renal biomarkers

Table 2 summarizes the levels of investigated markers of renal dysfunction at baseline and after 6 months of treatment. At baseline, there were no significant differences in the levels of renal biomarkers between the compared groups.

**Table 2:** Levels of renal biomarkers at baseline and 6 months after the beginning of the study in compared groups

Variable	Insulin-treated group (Control) n = 21		Insulin-vildagliptin-treated group (VIG) n = 23		p*	p**
	
	0 months	6 months	0 months	6 months		
Serum urea, mmol/L	5.6 [4.8; 6.5]	-	5.0 [4.0; 5.9]	-	0.08	NA
Serum creatinine, μmol/L	82.0 [74.0; 89.0]	80.0 [74.0; 90.5]	82.0 [71.0; 92.0]	84.0 [67.0; 94.0]	0.69	0.72
eGFRcreat, mL/min/1.73 m ^2^	77.0 [67.5; 84.4]	78.2 [65.3; 88.3]	69.8 [62.7; 95.0]	72.8 [61.8; 94.7]	0.62	0.86
Serum cystatin C, mg/L	0.87 [0.81; 1.01]	0.86 [0.79; 0.99]	**0.84 [0.76; 0.93]**	**0.75 [0.69; 0.82]**	0.12	0.01
eGFRcys, mL/min/1.73 m ^2^	89.9 [73.8; 100.8]	94.6 [71.5; 100.1]	**90.6 [81.9; 104.1]**	**100.9 [95.1; 107.6]**	0.29	0.049
eGFRcreat-cys, mL/min/1.73 m ^2^	80.0 [73.0; 91.0]	79.0 [66.5; 93.5]	**79.0 [75.0; 101.0]**	**90.0 [77.0; 104.0]**	0.44	0.18
Urinary albumin/creatinine ratio (UACR), mg/g Cr.	26.7 [20.1; 70.4]	27.2 [12.6; 89.1]	28.6 [15.0; 61.4]	24.0 [16.4; 47.3]	0.69	0.63
Urinary collagen type IV/creatinine (uCol IV/Cr), μg/g Cr.	3.76 [2.39; 7.15]	4.25 [2.48; 6.03]	**3.25 [1.72; 6.90]**	**1.73 [1.41; 4.12]**	0.29	0.21
Urinary NGAL/creatinine (uNGAL/Cr), μg/g Cr.	14.0 [5.3; 24.2]	16.6 [6.8; 27.9]	19.6 [8.6; 38.8]	15.7 [8.1; 38.3]	0.28	0.48

eGFRcreat: estimated glomerular filtration rate based on serum creatinine by CKD-EPI equation; eGFRcys: eGFRbased on serum cystatin C by CKD-EPI equation; eGFRcreat-cys: eGFR based on serum creatinine and cystatin C by CKD-EPI equation. – : not measured. NA: not applicable.

Data are reported as median [25th percentile; 75th percentile]. The difference with P<0.01 for comparison between the levels of renal biomarkers at baseline and 6 months after initiation of the study in the Wilcoxon signed-rank test is indicated in bold. p*– comparisons made between baseline values in the studied groups (by Mann-Whitney U-test). p** – comparisons made between studied groups by repeated measures in the General Linear Models (both measurements in each group are taken into account).

In the control group, the renal markers did not change significantly from baseline by the end of the study ( Table 2 ). In contrast, the reduction in uCol IV/Cr (-32.8 % [-55.8; -24.4], p = 0.001) and cystatin C level (-8.7% [-12,7; -3,6], p < 0.001) along with the increase in eGFRcys (+6.4 mL/min/1.73 m ^2^ [4.0; 10.9], p < 0.001) and eGFRcreat-cys (+4.0 mL/min/1.73 m ^2^ [1.0; 7.0], p < 0.001) from baseline values were obvious in the VIG group. At the same time, the decrease in UACR and uNGAL/Cr in vildagliptin-treated patients was not significant ( Table 2 ).

When we compared the repeated measurements of the studied markers in the VIG and the control groups, only the changes in serum cystatin C and eGFRcys differed significantly ( Table 2 ). Analysis of percentage changes in the studied parameters in the compared groups showed significant differences in the dynamics of serum cystatin C (1.0% [-3.9; 1.2] in the control group vs VIG, p < 0.001), eGFRcys (0.7% [-1.4; 4.3], p = 0.001, respectively), eGFRcreat-cys (1.5% [-3.0; 2.9], p = 0.005, respectively), and uCol IV/Cr (-2.0% [-13.5; 18.2], p < 0.001, respectively).

The results of correlation analysis showed that neither changes in serum cystatin C, eGFRcys, and eGFRcreat-cys nor changes in uCol IV/Cr. ratio in the VIG group were significantly related to the dynamics of fasting and postprandial glycemia as well as to the changes in HbA1c. We also found no factors associated with the dynamics of cystatin C, eGFRcys, and uCol IV/Cr. in stepwise regression analysis. However, an inverse association between the percentage change in systolic BP and eGFRcreat-cys was found (β = -0.47, R ^2^ = 0.22, p = 0.023). The history of cardiovascular disease in patients with T2DM, as well as smoking and current lipid-lowering and antihypertensive treatment, also did not have a noticeable impact on the dynamics of the significantly changed renal markers.

## DISCUSSION

In the current study including insulin-treated patients with satisfactory controlled T2DM, six-month administration of 50 mg vildagliptin daily improved glycemic and blood pressure control and reduced hsCRP level. Independently of hypoglycemic effect of the drug, renal effects of low-dose vildagliptin treatment manifested as a decrease in urinary excretion of type IV collagen and serum cystatin C level along with corresponding increase in eGFRcys and eGFRcreat-cys.

### The dynamics of metabolic and glycemic control parameters, blood pressure and hsCRP

In the VIG group we noted a significant reduction in HbA1c, frequency of hypoglycemic episodes along with insulin requirements, fasting and postprandial blood glucose levels from initial values. These findings are in line with the concept of the ability of the DPP-4 inhibitor vildagliptin to improve insulin and glucagon imbalance, thereby preventing development of hypoglycemia and decreasing the levels of fasting and postprandial glycemia (28,29).

Special attention should be paid to our findings of significant reduction in diastolic BP in the VIG group compared to the control. Pooled analysis by Evans et al. demonstrated the ability of vildagliptin to reduce both diastolic (from 81.2 ± 0.18 to 79.6 ± 0.19 mmHg, p < 0.0001) and systolic BP (from 132.5 ± 0.32 to 129.8 ± 0.34 mmHg, p < 0.0001) within 6 months of treatment even at the dose of 50 mg/day (30). Blood pressure is one of the most important clinical risk factors for the progression of DN. Vildagliptin could exert its mild antihypertensive effect at least partly through enhanced urinary sodium excretion and suppression of the renin-angiotensin system (31,32). Nevertheless, due to predominantly distal tubular natriuresis, DPP-4 inhibition does not affect tubuloglomerular feedback and, therefore, should not modify significantly renal hemodynamic function (33).

Our study did not find the weight loss effect of vildagliptin described by Evans and cols. (30). The use of low-dose vildagliptin combined with insulin therapy, an independent risk factor for weight gain (1,30), could be a possible explanation.

We also noted a significant reduction in hsCRP level after vildagliptin administration that is consistent with the findings of the study by Zografou and cols. (34) General population studies have shown a significant direct correlation between CRP levels and GFR deterioration (35).

### Changes in the levels of evaluated renal biomarkers

Along with other researchers we found no increase in creatinine-based eGFR during vildagliptin treatment (18,19,21). Consistent with the fact that DPP-4 inhibitors stimulate distal rather than proximal natriuresis (33,36), we did not observe any reduction in eGFRcreat as well. On the contrary, we found a significant increase in eGFRcys and eGFRcreat-cys in the VIG group. Serum cystatin C is a potential alternative to serum creatinine for estimating GFR that appears to be a stronger prognostic marker than creatinine (26,37). In T2DM patients, cystatin C reflects changes in glomerular filtration more accurately and rapidly than serum creatinine (38,39). Combined creatinine and cystatin C based eGFR-equation provides better precision and accuracy of GFR estimation than any of these markers alone with less diurnal variability (40,41) and regardless of the stage of CKD (26). The use of eGFRcys also seems more relevant, since cystatin C-based eGFR in routine clinical care was more closely associated with mortality compared to creatinine-based GFR estimation (42). A higher serum level of cystatin C is associated with inflammation, in particular, as assessed by CRP level (43). Our study demonstrated a significant decrease in hsCRP level in the VIG group. However, correlation analysis did not show any significant relations between the dynamics of hsCRP and cystatin C, eGFRcys and eGFRcreat-cys in the VIG group. Taking into account the above findings, we suggest that the observed increase in cystatin C-based eGFR could be considered a direct renal effect of vildagliptin. These results require confirmation in further population and experimental studies to clarify the mechanisms of observed increase in GFR after short-term treatment with vildagliptin.

We did not demonstrate any significant decrease in UACR on vildagliptin therapy contrary to other studies (18,19,21) uNGAL excretion is known to be elevated in early DN (24,44,45) and predictive of a rapid decline in eGFR (45). In this study, we did not observe significant changes in uNGAL/Cr in both groups. The lack of significant reductions in albumiuria and uNGAL could be partially explained by the use of a low dose of vildagliptin and the relatively short duration of the study. Indeed, experimental study by Liu et al. reported that vildagliptin decreased glomerular basement membrane thickening, tubule-interstitial fibrosis, and glomerulosclerosis in a dose- and time-dependent manner (15). Along with this, our study is the first to show a significant reduction in urinary Col IV/Cr excretion after 6 months of treatment with vildagliptin. Type IV collagen is a component released from glomerular and tubular basement membranes and mesangial matrix (24,25,44,46) that is positively correlated with the severity of mesangial expansion and glomerular injury in T2DM (46). uCol IV is known to be an earlier marker than albuminuria (44). We thereby interpret a significant decrease in urinary collagen IV excretion in the VIG group as an evidence of amelioration of early (preclinical) glomerular renal dysfunction in nonproteinuric type 2 diabetic patients. Importantly, identified renal effects of vildagliptin appear to be independent of its glucose-lowering effect, as no significant correlations with the decrease in HbA1C and blood glucose levels were found by correlation and regression analysis.

One of the limitations of our study is that our findings cannot be extrapolated to proteinuric T2DM patients, since our insulin-treated participants were at relatively early stages of diabetes-related CKD or without it. Relatively short duration of our observation, half-dose vildagliptin intake, and the small number of patients represent important limitations of the study. Finally, we were not able to verify DN by renal biopsies and to run direct evaluation of GFR by isotope clearance methods. However, to the best of our knowledge, this is the first study to assess in a particular way the effects of vildagliptin on preclinical markers of DN.

There is no doubt that in order to further investigate the revealed renal effects of vildagliptin we need long-term controlled clinical studies with assessment of hard endpoints (i.e. end-stage renal disease). Further studies are also required to investigate renal effects of vildagliptin in patients with various degrees of renal dysfunction.

In conclusion, this prospective randomized 6-month clinical study performed in insulin-treated patients with satisfactory controlled type 2 diabetes without overt CKD was the first to demonstrate the ability of vildagliptin added at the dose of 50 mg per day to reduce urinary type IV collagen excretion, serum cystatin C and to increase cystatin C-based eGFR and combined creatinine and cystatin C-based eGFR, in the absence of any significant influence on UACR. The study also found a significant reduction in HbA1c, incidence of hypoglycemic episodes along with a decrease in insulin requirements, diastolic blood pressure, and serum high-sensitivity C-reactive protein upon addition of vildagliptin as compared to continuation of insulin monotherapy in the control group.
